# Bayesian optimized indoor positioning algorithm based on dual clustering

**DOI:** 10.1038/s41598-024-79647-x

**Published:** 2025-03-18

**Authors:** Min Chen, Qiaolin Pu

**Affiliations:** 1 School of Communications and Information Engineering, Chongqing University of Posts and Telecommunications, Chongqing, China; 2 Department of Electronic Information and Communication Engineering, Chongqing Aerospace Vocational and Technical College, Chongqing, China

**Keywords:** Fingerprint database, Bayesian probabilistic algorithm, Fingerprint positioning, Dual clustering, Electrical and electronic engineering, Energy infrastructure

## Abstract

Wi-Fi indoor positioning provides a simple, convenient, ubiquitous and cost-effective solution by matching a pre-established Wi-Fi Received Signal Strength Indication (RSSI) fingerprint database with the RSSI values received from mobile terminals. However, due to the influence of the complex indoor environment on the signal, its accuracy can only reach the meter scale, and the huge fingerprint database leads to inefficient positioning. To solve this problem, the Canopy algorithm is used for coarse clustering, and then the K-means algorithm is used for fine clustering to determine the number of clusters and the initial clustering center to form multiple clustering sub-bases, which improves the positioning efficiency by about 95.05%. In the real-time matching stage, the sub-banks with the highest similarity are selected for matching by the correlation coefficient method, and combined with the Weighted K-Nearest Neighbors (WKNN) algorithm, this paper proposes an improved Bayesian probabilistic optimization algorithm, and the final experimental results show that the average positioning accuracy is improved by about 38.64%, the average runtime is shrunk by about 93.51%, and the stability of the system is slightly improved, which effectively improves the positioning accuracy, real-time performance, and stability.

## Introduction

Key technologies based on location services^1,2^ include location service platforms, areas of cooperation with location service platforms (public security, livelihood services, healthcare, Internet of Things, etc.), geographic information systems (GIS), network map services, search engine services, and heterogeneous network technologies to realize the interconnection and interoperability of the Internet and mobile communication networks.

However, as the complexity of the indoor environment increases, the scope of indoor positioning applications expands, and people’s demand for indoor activities increases, it is pointed out in the 2019 report “New Opportunities Brought about by Intelligent Positioning” released by AutoNavi that AutoNavi currently has more than 700 million users, and there are still many shortcomings in the existing positioning technology. In outdoor areas and indoor areas with more buildings and tunnels, satellite signals are obstructed, resulting in poor positioning accuracy^3^.

Reference^4^ used support vector machine regression to predict RSSI points of unknown points when building a Wi Fi fingerprint database. Due to the significant manpower and time costs invested in the database construction phase, machine learning algorithms need to collect a large amount of data in advance to construct a training set for subsequent training. Fingerprint recognition in reference^5^ usually takes a long time and is labor-intensive. It also requires a large amount of storage space. Minor changes in indoor environment may also require reassessment or even re memorization of RSSI values. The localization process in reference^6^ faces challenges such as spatial ambiguity, unstable RSSI, and short RSSI collection time.Yun Fen Yong^7^ proposed a new technique for constructing fingerprint maps based on Synthetic Minority oversampling (SMOTE) algorithm, which is used to generate synthetic fingerprints in areas that are difficult to reach or infrequently accessed in offline field investigations. The results show that although the accuracy decreases with the increase of synthetic data, it is still within an acceptable range of 0.64%; Xiaoqian Du^8^ proposed a crowdsourcing based wireless map construction method and trajectory matching algorithm to solve the time-consuming preliminary fingerprint data collection in the offline stage. In the offline stage, the tedious collection work is replaced by a large amount of crowdsourced sensor data. Real scene experiments show that this scheme can significantly reduce the time and manpower consumption of building wireless maps.

To address the problem of fingerprint matching and localization efficiency, JIN REN team^9^ proposed an improved high-precision public c-means (IPC) clustering algorithm for indoor environments and used it to optimize fingerprint databases.In the offline phase, a fingerprint database is built and clustered based on offline hybrid distance and affinity propagation clustering algorithms. This is because the signal strength tends to depend on the device used and the measurement time. Eventually, the sampled signals tend to form cluster groups relative to their acquired attributes.Sang Gu Lee’s team^10^ determined the optimal number of clusters and applied the K-means clustering algorithm to propose a more accurate algorithm for radio map generation. This process produces more accurate radio maps than the average sampling model.

In order to solve the above problems comprehensively, this paper proposes a Bayesian optimized indoor localization algorithm based on dual clustering under double clustering. Firstly, the offline fingerprint database is processed with improved Gaussian filtering; on this basis, the Canopy algorithm is used for “coarse” clustering, and then the K-means algorithm is used for “fine” clustering, and finally k clustered sub-bases are obtained. Finally, the sub-collections with the highest similarity are selected for matching by the correlation coefficient method. In the fingerprint matching stage, combining the traditional WKNN algorithm and the Bayesian probability algorithm, this paper proposes an improved Bayesian probability optimization algorithm to achieve the fingerprint matching, which can effectively improve the localization accuracy, real-time performance and stability.

## Fingerprint matching algorithm

The Wi-Fi fingerprint database positioning technology^11,12^ does not directly rely on the distance or angle relationship between the test point and the wireless transmission node. It establishes a relationship between the RSSI^13,14^ of the AP node received by the current test point and the RSSI at a certain position.

The RSSI at a certain position is the signal strength value related to the position information collected by the reference point at this position. RSSI can reflect the multipath characteristics of radio signals at various positions, and the physical characteristics it exhibits are called “fingerprints”, that is, the RSSI at a certain position can be called a certain fingerprint point^15,16^. Location information can be obtained through geographic location maps and indoor floor plan coordinate maps, and collecting the RSSI^17^ sequences of each AP node obtained from the current location coordinates, a location fingerprint database can be constructed by collecting all the location fingerprint points. Finally, the current position coordinates of the test point are indirectly obtained by constructing an RSSI corresponding to a certain position information in the location fingerprint database.

**Figure 1:**
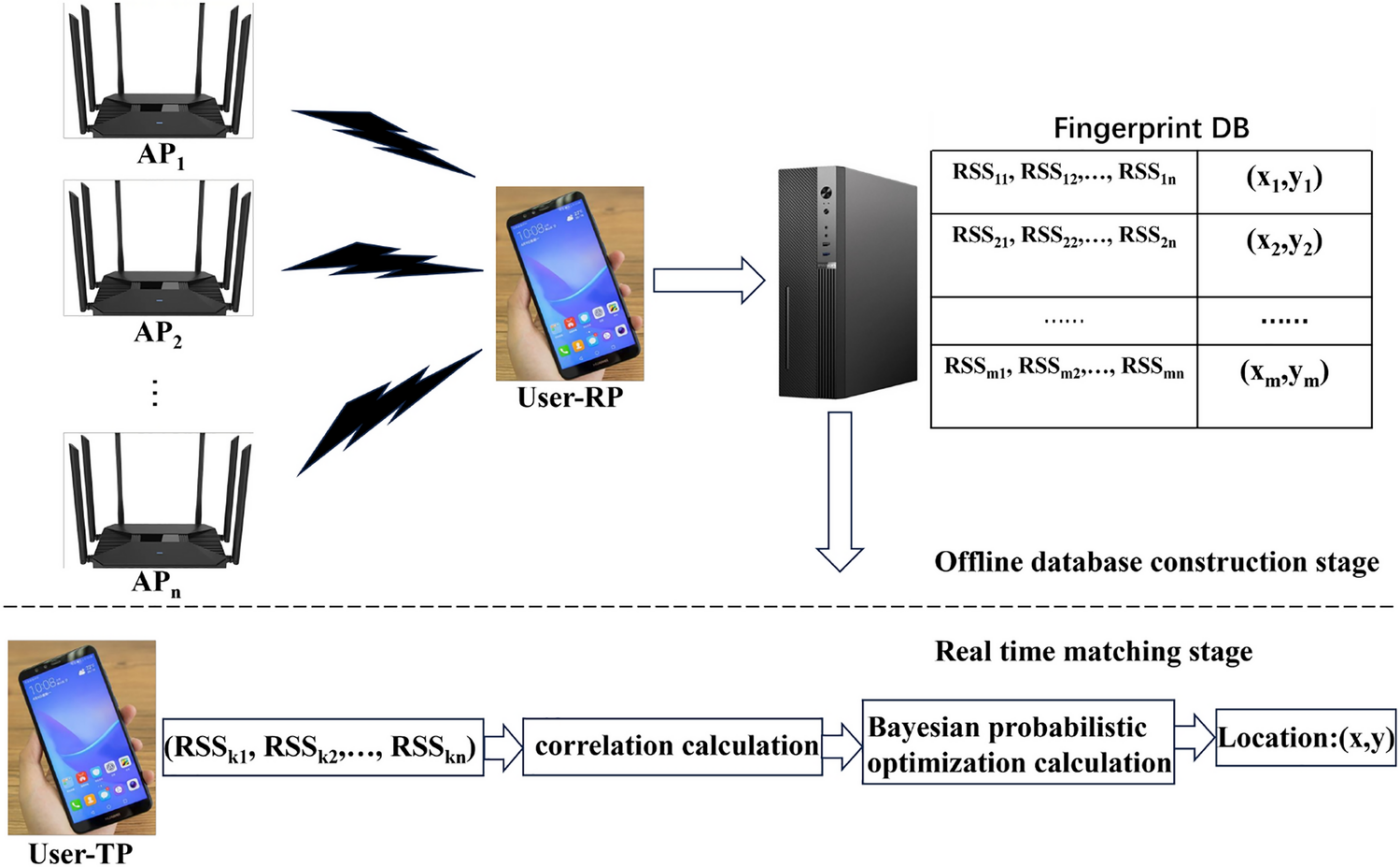
Fingerprint matching positioning principle.

The indoor positioning method using fingerprint matching^18,19^ is to obtain the position coordinates of the current test point by calculating the correlation between the RSSI of the test point and the RSSI of the location fingerprint database, Fig. 1 shows the principle of fingerprint matching and localization. Therefore, it is usually divided into: 1) Offline database construction stage: Set fingerprint points on indoor planar coordinate maps or geographic location maps, and select enough fingerprint points on the map^20,21^ for data collection. Establish an offline location fingerprint database based on the RSSI sequence and fingerprint location information collected from the fingerprint points. 2) Real time matching stage: The correlation between the RSSI sequence received by the test point and the RSSI sequence of the location fingerprint database is calculated, and then the position information corresponding to the fingerprint points in the location fingerprint database is calculated as the position coordinates of the test point^22,23^.

In the real-time matching stage, the selection of fingerprint matching algorithms mainly affects positioning accuracy. Fingerprint matching algorithms^24–26^ can be divided into deterministic matching algorithms and probabilistic matching algorithms based on principles.

The deterministic matching algorithm is based on the position coordinates of the fingerprint points^27–29^ adjacent to the test point and the RSSI sequence, and finally obtains the position coordinates of the test point through deterministic estimation, such as the proximity method algorithm, correlation coefficient method, and support vector machine. Probabilistic matching algorithms typically use Bayesian inference to estimate the position coordinates of the target point, such as naive Bayesian algorithms.

## The proposed Bayesian optimized indoor positioning algorithm based on dual clustering

In this section, a Bayesian optimized indoor localization algorithm based on dual clustering is presented. First, we outline the use of improved Gaussian filtering algorithm for denoising. Then the dual clustering algorithm is introduced to cluster the fingerprint library, and finally the localization is achieved by the Bayesian probabilistic optimization algorithm.

A.RSSI Noise Reduction Processing

The overall RSSI sample data shows a Gaussian distribution trend, with the highest probability occurring when the RSSI is -52dBm, and the probability value gradually decreases as it extends to both sides. To better fit the distribution curve of RSSI sample data, this paper introduces a Gaussian distribution model, assuming that the RSSI sample data obeys $(u,s^{2})$ Gaussian distribution, and the probability density function formula is shown in Eq. (1).

1 $$\begin{aligned} f(rssi) = \frac{1}{s\sqrt{2\pi }} e^{-\frac{(rssi-u)^2}{2s^2}} \end{aligned}$$

among

2 $$\begin{aligned} u= & \frac{1}{N} \sum _{i=1}^N r s s i_i \end{aligned}$$

3 $$\begin{aligned} s= & \sqrt{\frac{1}{N-1} \sum _{i=1}^N\left( r s s i_i-\frac{1}{N} \sum _{i=1}^N r s s i_i\right) ^2} \end{aligned}$$

In the formula, u is the mean, s is the standard deviation, N is the number of RSSI sample data, and rssii is the RSSI sample data collected for the i-th time.

For small probability events with significant fluctuations caused by noise interference, Gaussian filtering is used to filter out small probability events, which may lead to effective information loss. Therefore, this article improves Gaussian filtering, and the specific process is as follows:

(1) Input RSSI sample data: To reduce noise interference, a four-way multiple acquisition method is adopted during the collection of RSSI sample data.

(2) Gaussian filtering^30^: By introducing a Gaussian model to filter the RSSI sample data collected in the first step, this paper selects a high probability interval with a probability value of about 90%, that is, RSSI$\in$(u-1.65s, u+1.65s), where u is the mean and s is the standard deviation.

(3) Low probability RSSI sample data: Use the second step of Gaussian filtering to remove low probability points. Set the low probability sample set to T and the number of samples to k, T=[$RSSI_{out1}$, $RSSI_{out2}$,..., $RSSI_{outk}$].

Assign values to the RSSI sample data in set T as shown in Eq. (4).

4 $$\begin{aligned} R S S I_{\text{ outj } }=\frac{1}{N-k} \sum _{i=1}^{N-k} R S S I_i, j=1,2, \ldots , k \end{aligned}$$

In the formula, RSSIoutj represents the sample data in the sample set T, and N represents the total RSSI sample data.

(4) Output RSSI noise reduction data: After assigning values to the low probability sample data, it is merged into the high probability sample data to complete the RSSI noise reduction process for the current fingerprint point, and the RSSI data is saved to the offline fingerprint database.

Smooth mean filtering is based on mean filtering based on optimization, in the data buffer according to the order of the same fingerprint point of the N RSSI sample data, in accordance with the “first-in-first-out” stack idea to keep the total length of the data buffer is unchanged, through the calculation of the data buffer RSSI data mean and as the current position of the RSSI value. The average value of RSSI data in the data buffer is calculated and used as the RSSI value of the current position.

5 $$\begin{aligned} RSSI=\frac{1}{N}\sum _{i=1}^{N}RSSI_{i} \end{aligned}$$

**Figure 2:**
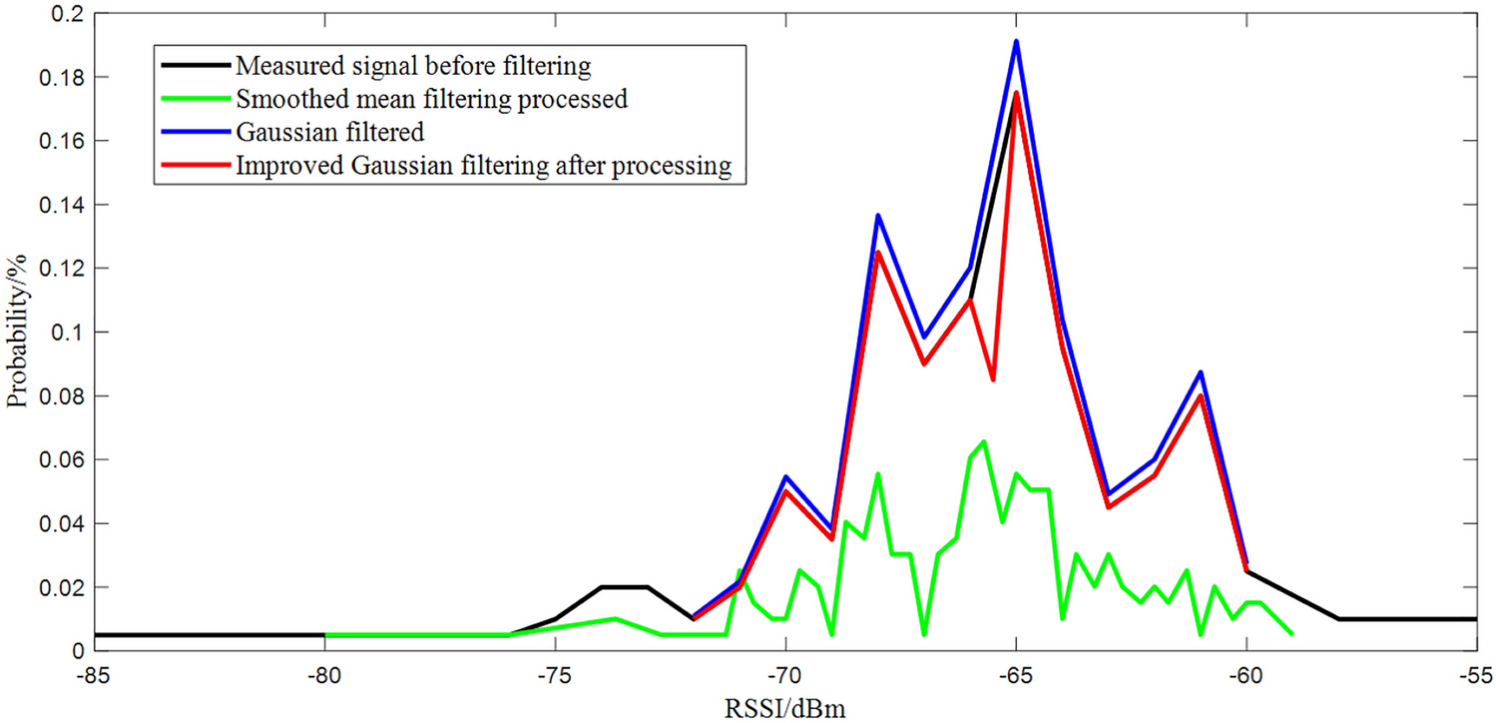
Comparison chart before and after RSSI filtering.

**Figure 3:**
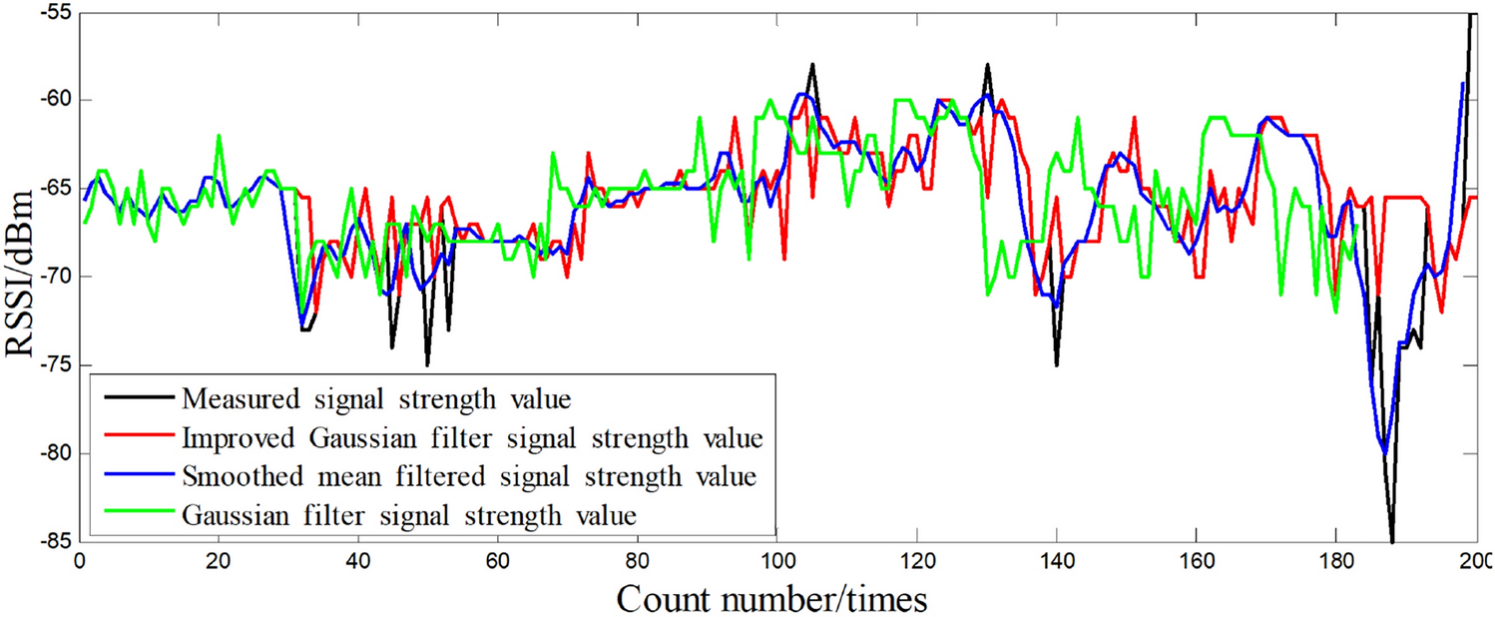
Comparison chart before and after RSSI filtering.

Comparing the two methods of smoothing mean filtering and Gaussian filtering, Fig. 2 shows the distribution comparison before and after RSSI filtering, Fig. 3 is a comparison diagram before and after RSSI filtering.

B.Evaluation of clustering effects

(1) Elbow method:The core indicator of the elbow method is the sum of squared errors E. As the number of clusters^31^ k increases, the fingerprint database partition will become more refined, and the degree of aggregation of each cluster will gradually increase, resulting in a gradual decrease in the sum of squared errors E. The formula for calculating the sum of squared errors is shown in Eq. (6).

6 $$\begin{aligned} E=\sum _{j=1}^k\sum _{RSSI_i \in C_j}\left( \text {RSSI}_i-\text {Center}_j\right) ^2 \end{aligned}$$

In the formula, k represents the given number of clusters, RSSI_*i*_ represents all fingerprint points in the current j-th sub database except for the cluster center, and Center_*j*_ represents the cluster center of j-th class.

(2) Silhouette Coefficient method:The silhouette coefficient method uses the core indicator silhouette coefficient SC to determine, combined with the silhouette coefficient calculation formula for clustering effectiveness indicators, to calculate the corresponding k value when selecting a larger coefficient. The range of silhouette coefficient values is between [$-1$,1], and it can be used as a measure to determine whether the clustering effect is reasonable and effective. The larger the silhouette coefficient value, the more reasonable the clustering effect.

**Figure 4:**
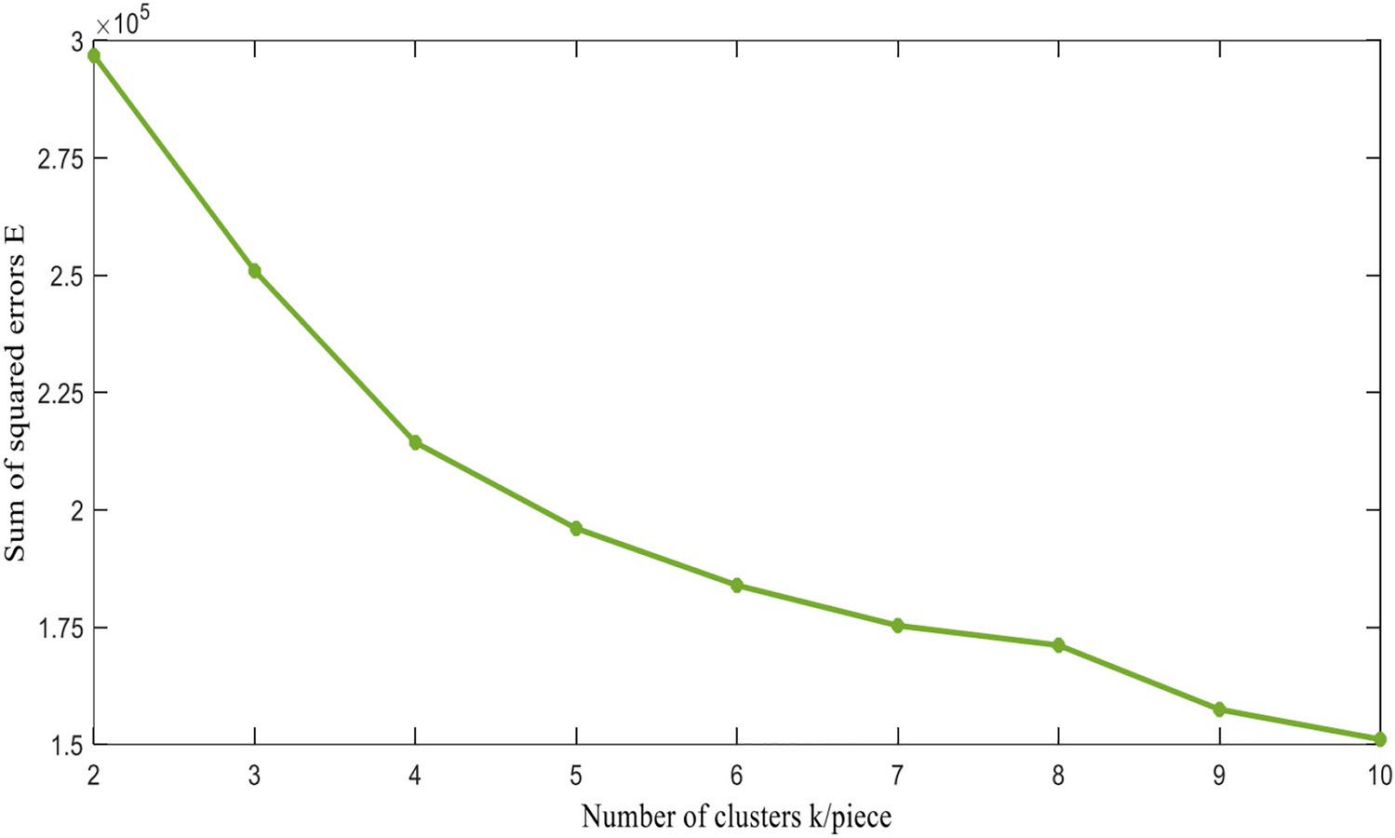
Relationship between the sum of squared errors E and the number of clusters k.

**Figure 5:**
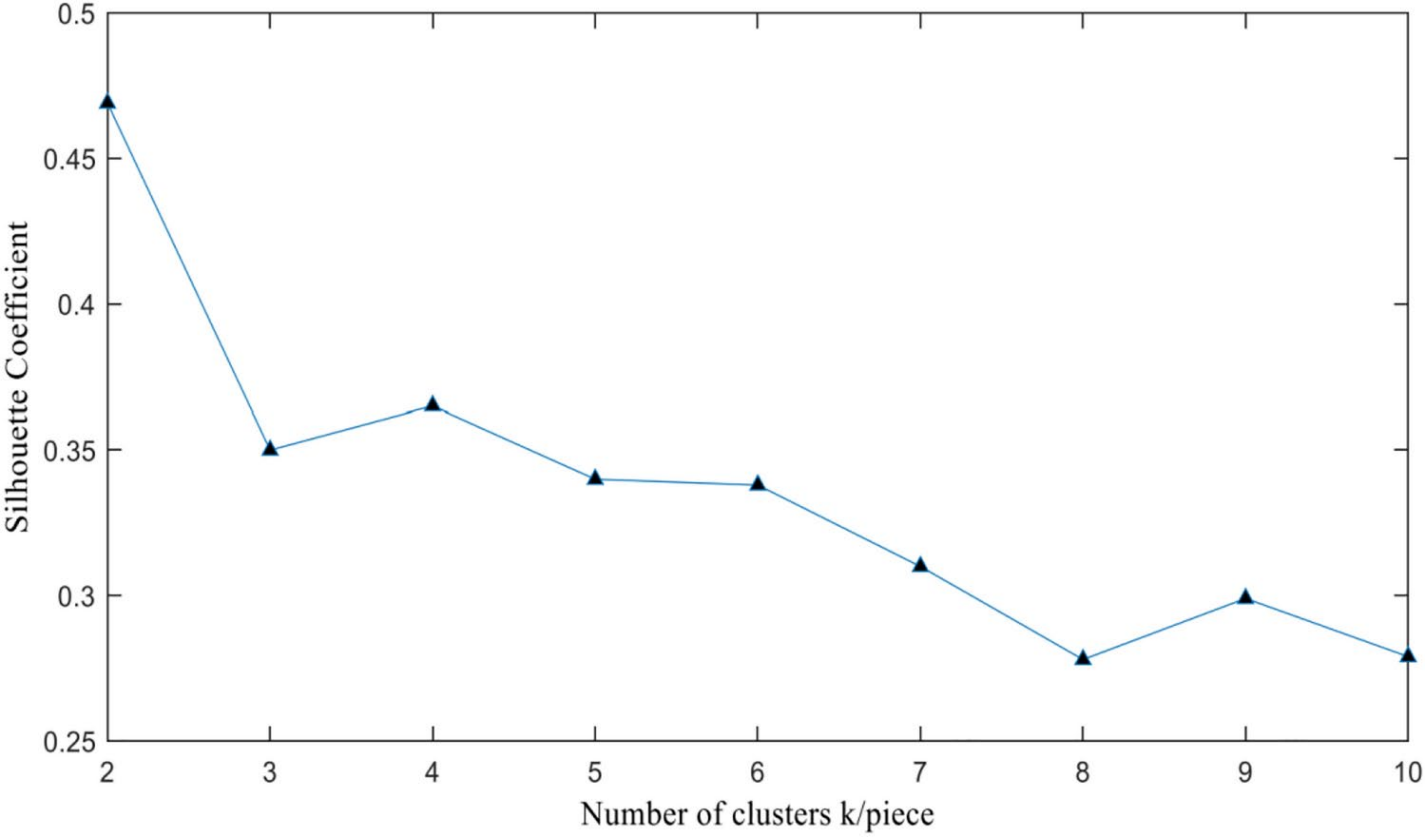
Relationship between silhouette coefficient SC and number of clusters k.

Using the K-means algorithm for clustering, record the sum of squared errors E and the contour coefficient SC values as the number of clusters k increases one by one, and draw the relationship graphs between k, E, and SC. Figure 4 shows the relationship between the number of clusters k and the sum of squared errors E. It can be seen from the relationship graph that when k = 6, it is located at the elbow position of the elbow graph. k = 6 is the true number of clusters in the data, which means the optimal number of clusters in this experiment is 6. Figure 5 shows the relationship between the number of clusters k and the contour coefficient SC. When k $>=$ 6, the overall value of the sum of squared errors is small. At the same time, considering that the larger the average contour coefficient, the better the clustering effect, the analysis and final determination of the number of clusters k = 6.

C. Double clustering

Firstly, the K-means algorithm is susceptible to the influence of singular points, while in the optimization process of the fingerprint database in the third section, singular points are removed through Improved Gaussian filtering . How to reasonably select the number of clusters^32–34^ k in the K-means algorithm, the optimal k value is determined by combining the sum of squared errors E with the contour coefficient SC. Considering that the K-means algorithm must determine the initial cluster center C before clustering, and usually the initial cluster center is randomly given from the dataset, if the initial cluster center selection is not ideal, it will directly affect the final clustering effect.

**Figure 6:**
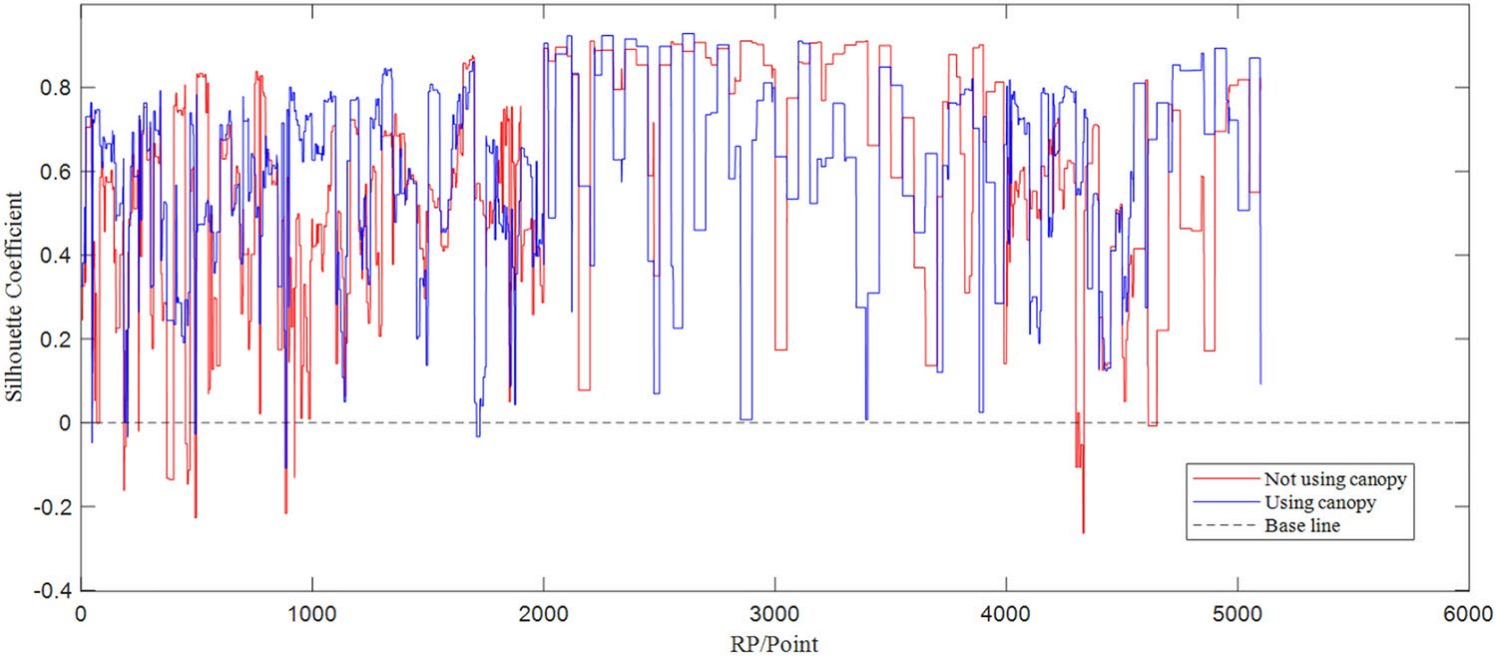
Comparison of Silhouette coefficient.

**Figure 7:**
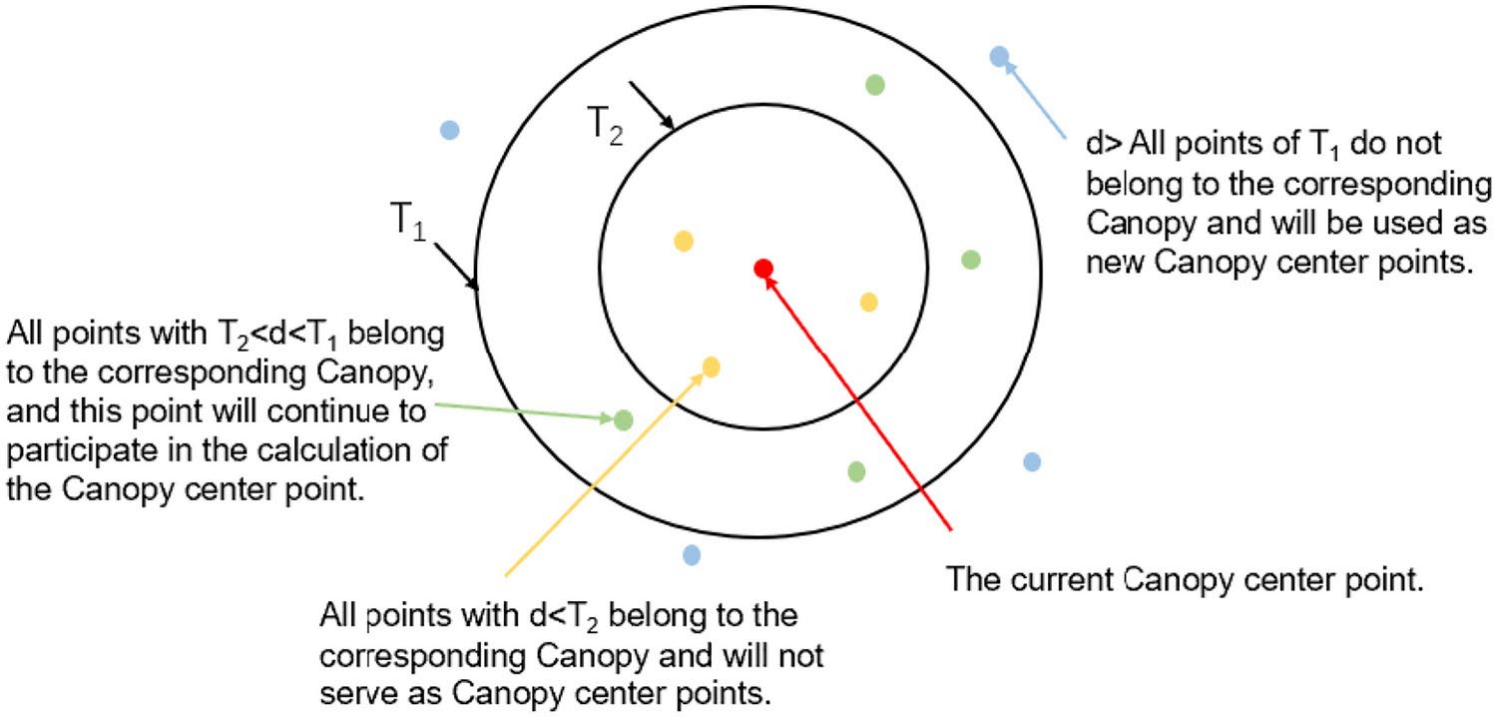
Clustering process of a single Canopy center point.

The experimental data was sourced from the fifth floor of the third teaching building at Chongqing University of Posts and Telecommunications^35^. The data was validated through K-means clustering of uncovered and covered datasets. The clustering effect is measured by the silhouette coefficient (SI), sum of squared errors (SSE). In Fig. 6, after coarse canopy clustering, the overall SI value is high, indicating good clustering effect. Meanwhile, after double clustering, the SSE is $3.1594 \times 10^5$, while after K-means clustering, the SSE is $4.9338 \times 10^5$. This indicates that the dual clustering effect proposed in this article is more significant. In the preprocessing stage, the Canopy algorithm is typically used to perform “coarse” clustering on fingerprint databases to determine initial clustering centers, and then further process the data based on the “coarse” clustering results. Figure 7 shows the clustering process of the Canopy algorithm when a single Canopy centroid appears.

The specific steps of the Canopy algorithm are as follows:

1. Given a List of N fingerprint points that have undergone RSSI denoising, set the distance thresholds T_1_ and T_2_ for Canopy, where T_1_>T_2_;
2. Randomly select a point p from the fingerprint database List, and use p as the first Canopy center point, while removing this point from the List;
3. Continue to select a point from the List and calculate the distance d from that point to all generated Canopy center points. If the distance d (d$>=$T_1_) from this point to all Canopy center points, then the point will be considered as the new Canopy center point and removed from the List; If the distance d (T_2_<d<T_1_) between the point and the center point of a Canopy, the point will be added to the Canopy and will continue to participate in the calculation; If the distance d (d$<=$T_2_) from a Canopy center point at that point between the center point of a Canopy at that point is, then add that point to the Canopy and remove it from the List;
4. Repeat step (3) until the List is cleared;
5. Ultimately, k Canopy center points and k Canopies are generated.

Canopy+K-means dual clustering: This section will integrate the Canopy algorithm and K-means algorithm to form a complete dual clustering optimization algorithm. The specific steps of the dual clustering algorithm are as follows:

(1) Using the RSSI denoising method proposed in Chapter 3, preprocess the fingerprint database to remove singular points and obtain high-quality fingerprint database data RSSI, $FP = \{(RSSI_{i}, (x_{i}, y_{i}))\}_{i=1}^{N}$.

Where FPi is the RSSI information received from all AP nodes at the i-th fingerprint point;

(2) Calculate the correlation between the sum of squared errors E in the elbow method and the contour coefficient SC in the contour coefficient method, and comprehensively analyze to determine the optimal value of k;

(3) Take the preprocessed fingerprint database data as input data to form a List, and set the distance thresholds T_1_ and T_2_ for Canopy. Using the Canopy algorithm to achieve “coarse” clustering, k initial cluster centers were obtained,$Center = \{Center_{1}, Center_{2}, \ldots , Center_{k}\}$;

(4) Take the initial cluster center generated in step 3) as the input of the K-means algorithm, use the K-means algorithm to achieve “fine” clustering and update the intra class data, and use the result at the end of the iteration condition as the final clustering result. At this time,$Center' = \{Center_{1}', Center_{2}', \ldots , Center_{k}'\}$.The intra class situation is $C_{i}={(RSSI_{ij},(x_{ij},y_{ij}))}_{j=1}^{q}$.

The number of fingerprint points is q in the i-th sub database.

**Figure 8:**
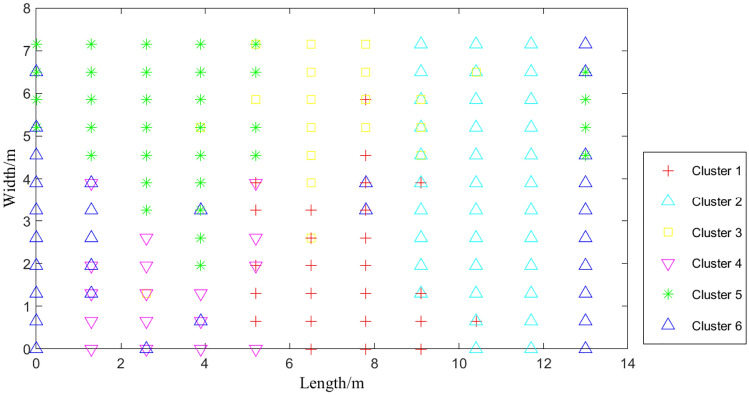
Planar effect after clustering with k = 6.

To avoid the impact of the number of clusters on the results during the clustering process, the maximum number of clusters is set to 100, and the automatic clustering results are shown in Fig. 8.

**Figure 9:**
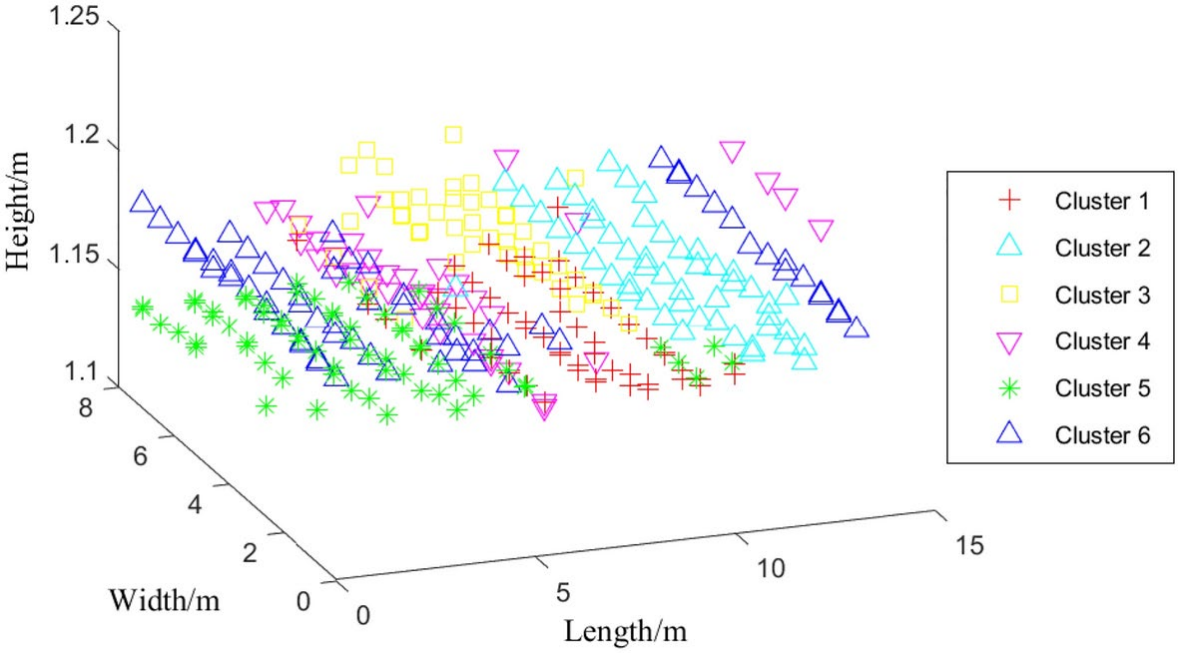
Stereoscopic effect after clustering with k = 6.

From Fig. 8, it can be seen that multiple sets of data at the same location may be divided into different clusters, and verifying the same location requires collecting data from multiple directions; Fig. 9 shows the three-dimensional effect of clustering, which clearly shows that the distribution of each cluster is relatively uniform, verifying the good clustering effect.

D.Bayesian optimized algorithm

Since the Bayesian probabilistic algorithm needs to calculate the posterior probability corresponding to each reference point when estimating a single mobile position, it requires a large number of computations, which leads to a large delay. To solve this problem, we propose a Bayesian probabilistic optimization algorithm that integrates WKNN^36^ into the Bayesian probabilistic algorithm to reduce the computational burden and improve the positioning accuracy^37–40^. In the offline database building phase, we select M reference points, measure the RSSI signals of N access points at these points, and build them into a set S,$\{S_{1}, S_{2}, \ldots , S_{m}\}^{T}$. The data set of the i-th reference point is $\{{(S_{i1}, S_{i2}, \ldots , S_{im}), (x_{i},y_{i})}\}$, where $(S_{i1}, S_{i2}, \ldots , S_{im})$ denotes the measured signal set S, and $(x_{i}, y_{i})$ denotes the current position $L_{i}$ of the ith RP.In the position matching stage, the kth position to be measured (RSSI data is recorded as $S_{k} = [S_{k1}, \ldots , S_{kj}, \ldots , S_{kn}], k \in N^{+}$, is at the following distance from any jth reference point in the fingerprint database:

7 $$\begin{aligned} D_{i} = \left( \sum _{j=1}^{n} |S_{kj} - S_{ij}|^{q}\right) ^{1/q}, i = 1, 2,\ldots ,m \end{aligned}$$

where, q = 1 stands for Absolute Distance; q = 2 stands for Eucleadian. here, we choose q = 2. the smaller the value of $D_{i}$, the higher the similarity between two points.

Using Euclidean distance^32^, we first calculate the T numbers of $D_{k}'s$, $D_{k} = [D_{1}, \ldots , D_{i}, \ldots , D_{m}]$, and then sort them in ascending order, $\{{(S_{i1}, S_{i2}, \ldots , S_{im}), (x_{i}, y_{i})}, i<l+1\}$, and then use WKNN to select the first $(t-1)'s$ nearest neighbors, $\{ (S_{i1}, S_{i2}, \ldots , S_{im}), (x^{\prime }_{i}, y^{\prime }_{i})\}, i<l+1$. The final position coordinates are calculated by using Bayesian probabilistic optimization algorithm.

The ith Reference Point (RP) in the fingerprint library receives the Access Point (AP) RSSI signal as $S_{j}=[S_{i1},\ldots , S_{ij},\ldots , S_{in}], j=1,\ldots ,n$,where the number of RPs is m and the number of APs is n. Constructing the fingerprint database $S_{i}=[S_{i1},\ldots , S_{ij},\ldots , S_{in}], i=1,\ldots ,m$, where the location information $L_{i} = (x_{i}, y_{i})$ of the ith reference point is calculated by Eq. (8) calculates the a posteriori probability of the occurrence of the real signal at the ith reference point denoted as P(L_i_/S), i.e:

8 $$\begin{aligned} P(L_i / S) = \frac{P(S/L_i)P(L_i)}{\sum _{i=1}^{m}P(S/L_i)P(L_i)} \end{aligned}$$

where P(S/L_i_) is the conditional probability that the ith reference point receives the real measurement signal S at the location, and P(L_i_) is the probability event of the occurrence of the location information at the first reference point, and it is generally considered that the probability of the occurrence of the location information at any reference point is equal, i.e., it obeys the uniform distribution P(L_i_) = 1/m. Assuming that the real measurement signals S emitted by the n access points at the ith reference point are all independent of each other and do not interfere with each other, i.e:

9 $$\begin{aligned} P(S/L_{i})=P(s_{1}/L_{i})P(s_{2}/L_{i})P(s_{n}/L_{i}) \end{aligned}$$

From the relevant theory of probability statistics, it can be concluded that any ith to-be-measured point receives the actual measured signal of the jth access point signal $s_{j}$ all satisfy Gaussian normal distribution,where u,sigma respectively, is the mean and standard deviation of the measured signal strength of the jth access point received by the ith reference point, i.e:

10 $$\begin{aligned} P(s_j / L_i)=\frac{1}{\sqrt{2\pi }\sigma } \exp \left[ -\frac{(s_j - u)^2}{2\sigma ^2}\right] \end{aligned}$$

Finally P(L_i_/S) is used in the target localization area as the weight coefficient of L_i_ position information corresponding to the ith reference point, and the final position information $(\widehat{x}, \widehat{y})$ is estimated by Eq. (11), i.e:

11 $$\begin{aligned} (\widehat{x},\widehat{y}) = \sum _{i=1}^{m}p(Li/S)(x_{i}^{\prime }, y_{i}^{\prime }) \end{aligned}$$

**Figure 10:**
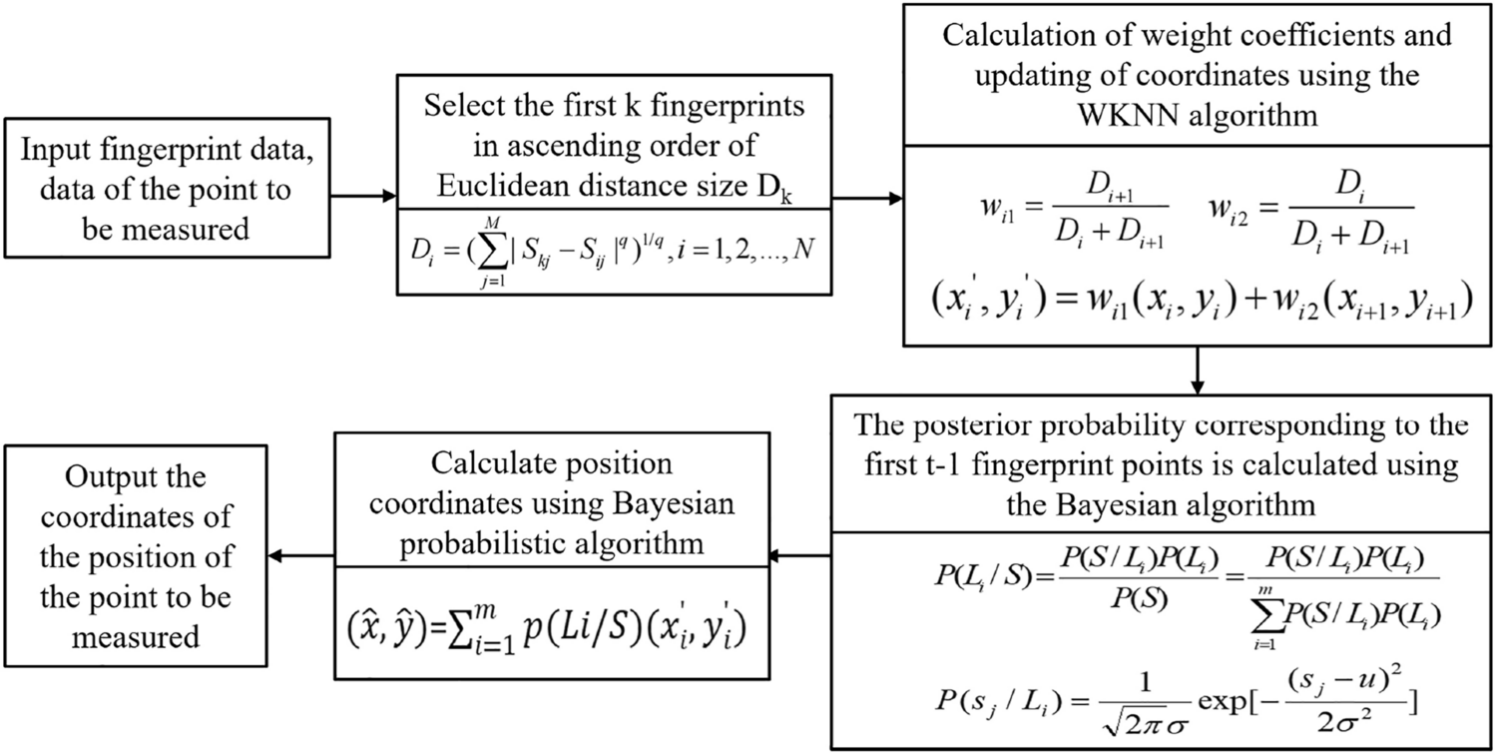
Bayesian probabilistic optimization algorithm processing flow.

Figure 10 shows the processing flow of Bayesian probabilistic optimization algorithm.

**Algorithm 1:**
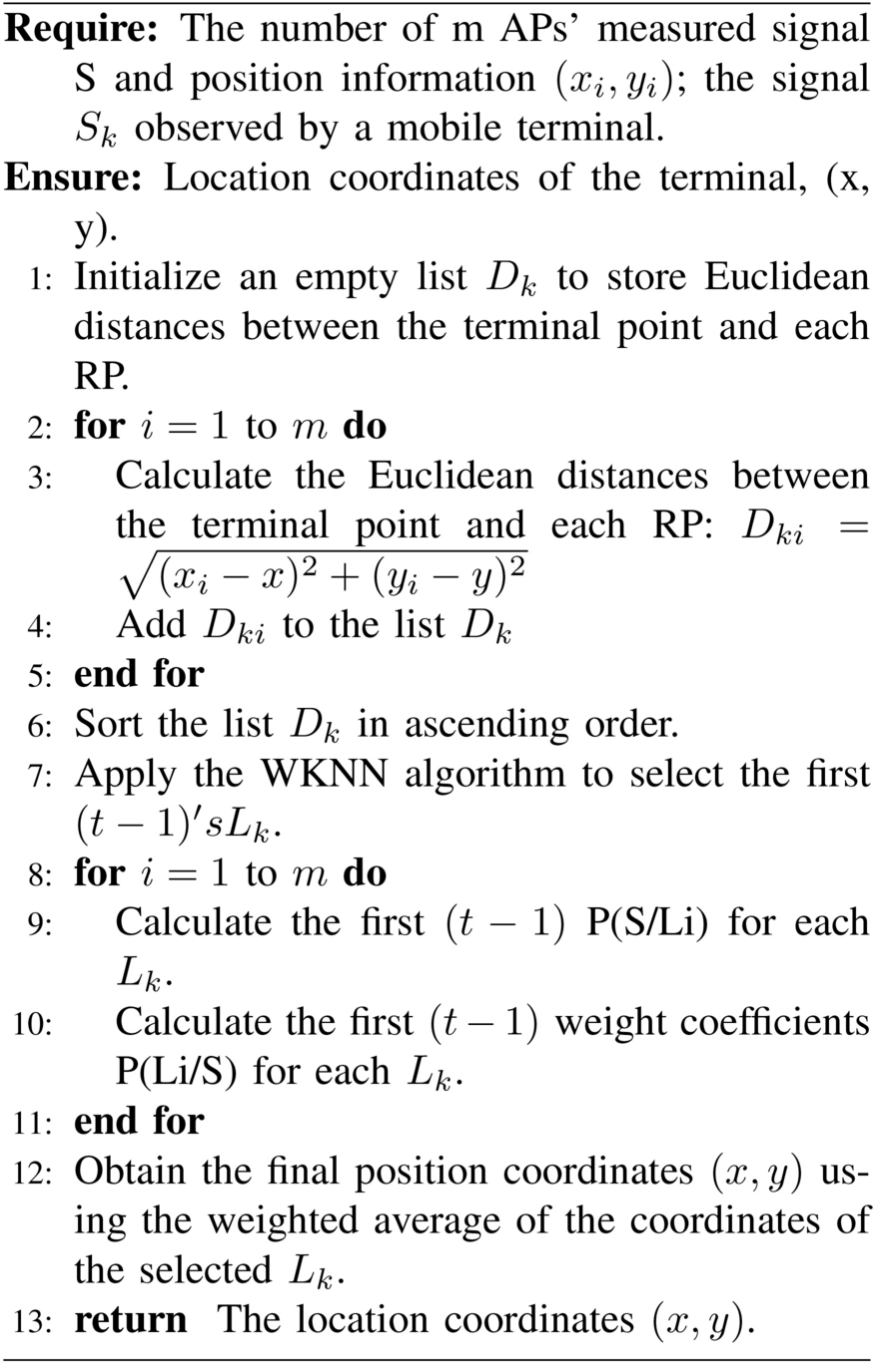
Location Estimation Algorithm

## Experimental results

**Figure 11:**
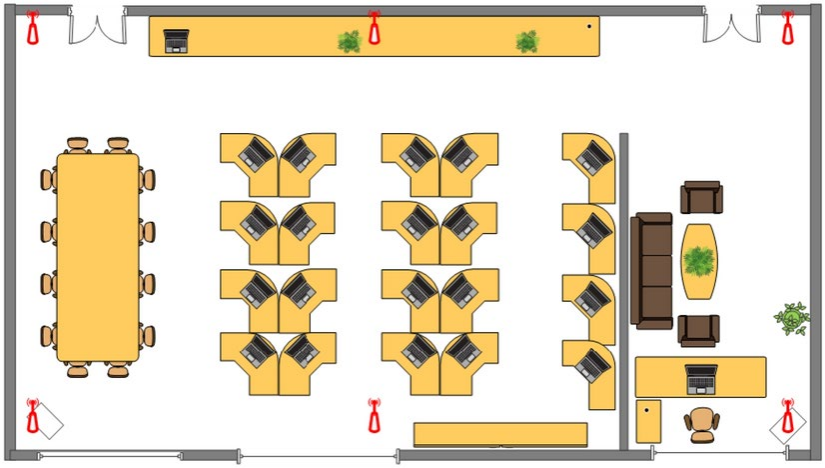
Experimental environment AP node layout plan.

This paper, a typical laboratory environment is selected as the indoor localization area, and in order to facilitate the analysis of the number of AP nodes and deployment locations, the laboratory localization area is transformed into a localization floor plan, as shown in Fig. 11. This experiment uses a TP-LINK wireless router as the AP node and Huawei EMUI 9.1.0 as the RP fingerprint point collector, and uses the test points for subsequent testing.

A.Bayesian optimized indoor positioning algorithm effect

By comparing the experimental results of traditional WKNN, Bayesian probabilistic algorithm and Bayesian probabilistic optimization algorithm, the advantages of the Bayesian probabilistic optimization algorithm proposed in this paper are verified by using the parameters of average positioning error, standard deviation of the error, and average running time to illustrate the positioning accuracy, positioning stability, and real-time performance of the positioning of each algorithm.

**Figure 12:**
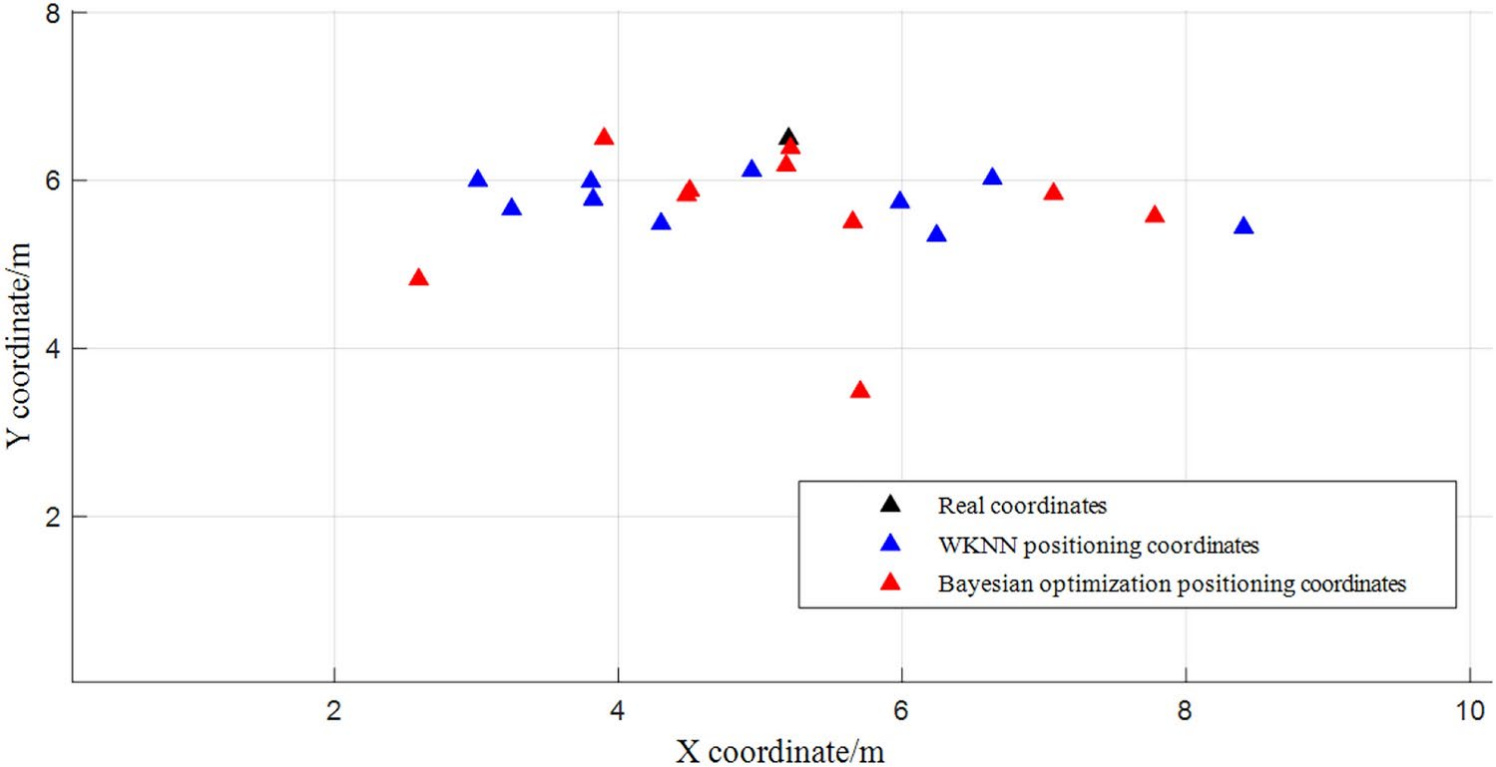
Comparison of the optimized algorithm for positioning the point to be measured $p_{2}$:(5.2,6.5).

**Figure 13:**
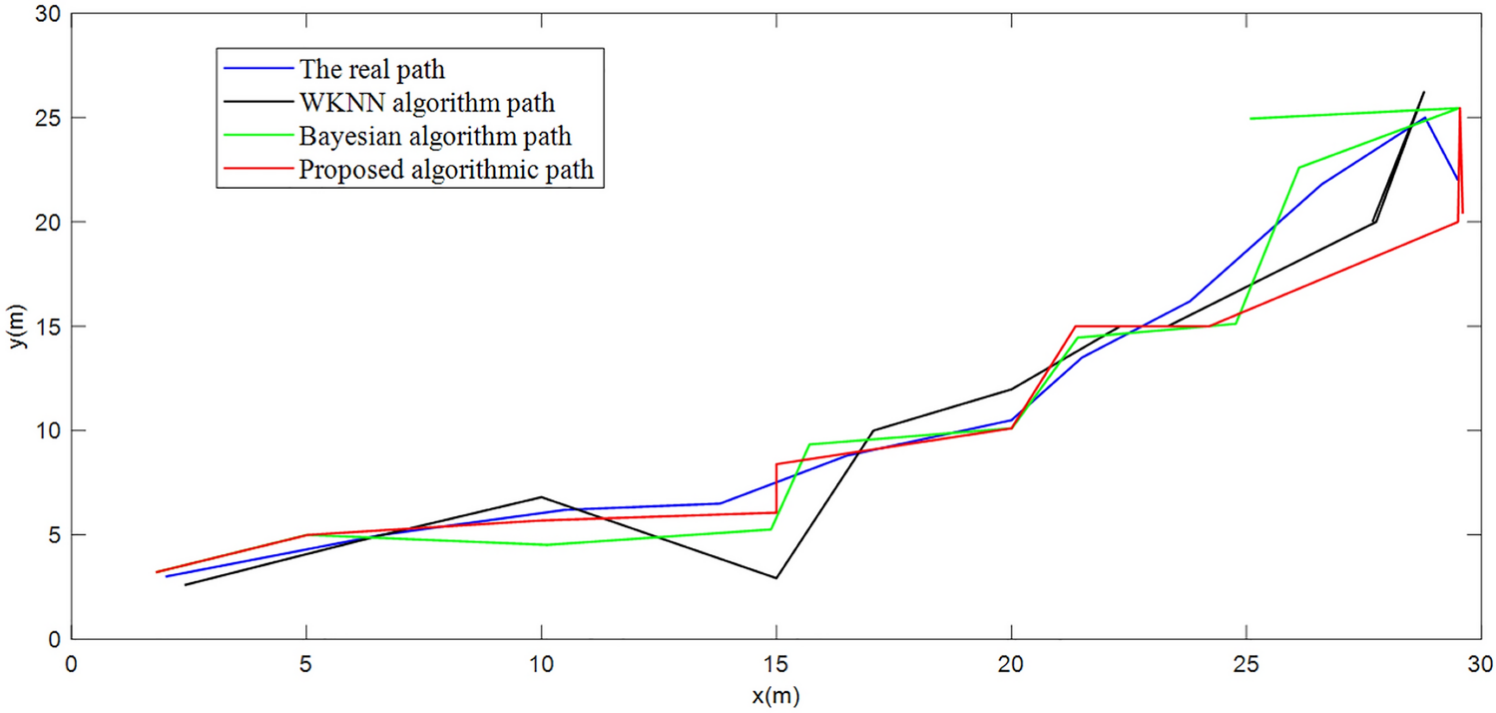
Positioning path comparison.

Considering the randomness of the proposed algorithm, 10 sets of RSSI data are collected from the same location of $p_{1}$ and $p_{2}$ and tested by WKNN, Bayesian probability algorithm and Bayesian probability optimization algorithm. RSSI data are collected at the same location of the current point $p_{1}$ and $p_{2}$, and the WKNN, Bayesian probabilistic algorithm and Bayesian probabilistic optimization algorithm are used for the location test and path test. The localization results are shown in Figs. 12 and 13.

In the above test, the real coordinates of the two points to be measured were compared with the positioning coordinates matched by each algorithm, and the positioning coordinates under the Bayesian probabilistic optimization algorithm were closer to the real coordinates and did not have a large deviation when the device was at a standstill state compared to the WKNN and Bayesian probabilistic algorithms.

B. Effectiveness of Bayesian optimized indoor positioning algorithm based on dual clustering

**Figure 14:**
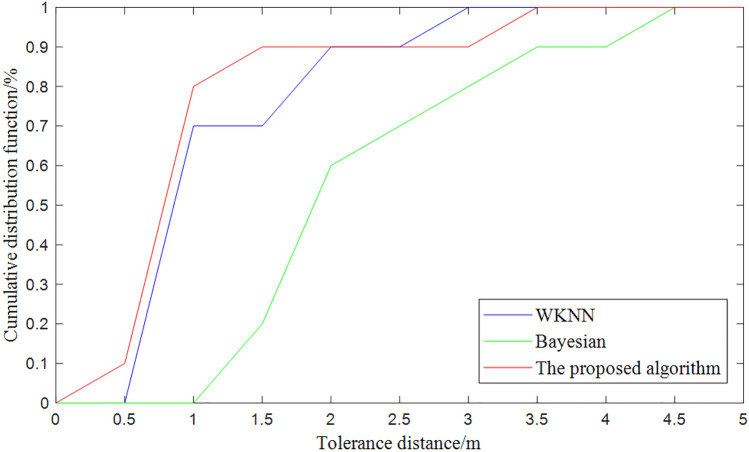
Error curves of localization results based on double clustering.

Compare the experimental results of WKNN, Bayesian and Bayesian optimization algorithms on the basis of Canopy+K-means dual clustering. Taking 20 mutually unrelated to be tested points for testing, the specific experimental results are shown in Table 1, and Fig. 14 shows the error curves of the localization results of the three algorithms.

**Table 1:** Clustering experiment results.

Parameter	Mean squared error	Operation time(s)	Accumulated error probability(%)					
			0.5m	1m	1.5m	2m	2.5m	3m
No clustering	0.7370	4.4222	9.8	10.02	39.52	70.11	87.75	90
K-means(WKNN)	0.9383	0.3295	10	29.9	50.1	75.54	90	100
Canopy+K-means (WKNN)	0.8033	0.2188	15.23	39.72	51.14	80.20	90	100
Canopy+K-means (Bayesian)	0.9156	0.6171	0	10.5	20.0	61.5	70.4	81.2
The algorithm proposed	0.7214	0.2872	10.0	80.5	90.5	91.0	92.4	92.4

Compared with the initial WKNN average positioning error of 1.6758 m, under the final improvement: the average positioning error is 1.0282 m, the average positioning accuracy is improved by about $38.64\%$, and the average running time is reduced by about $93.51\%$.

**Figure 15:**
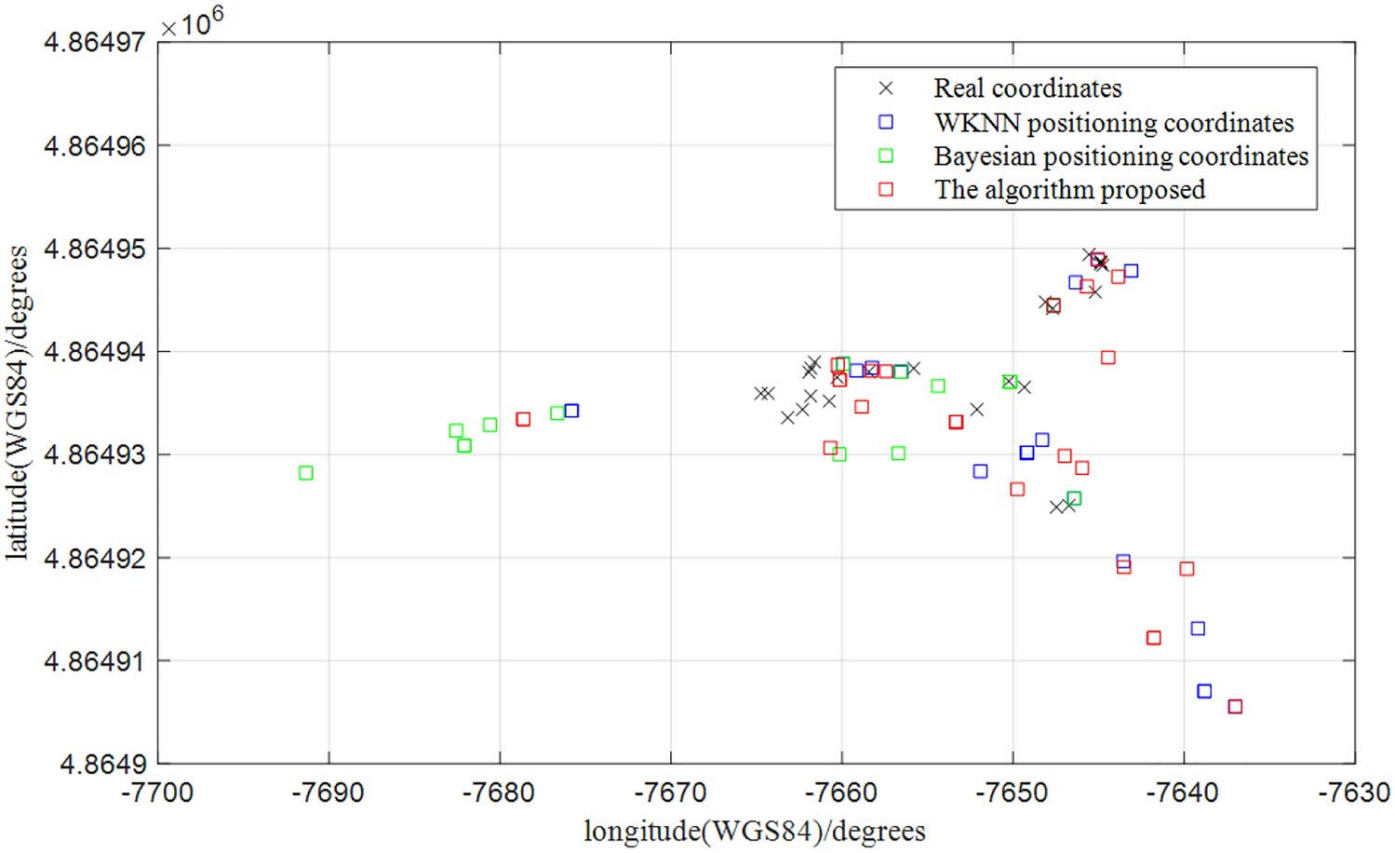
Comparison of the optimized algorithm for positioning the point to be measured.

**Figure 16:**
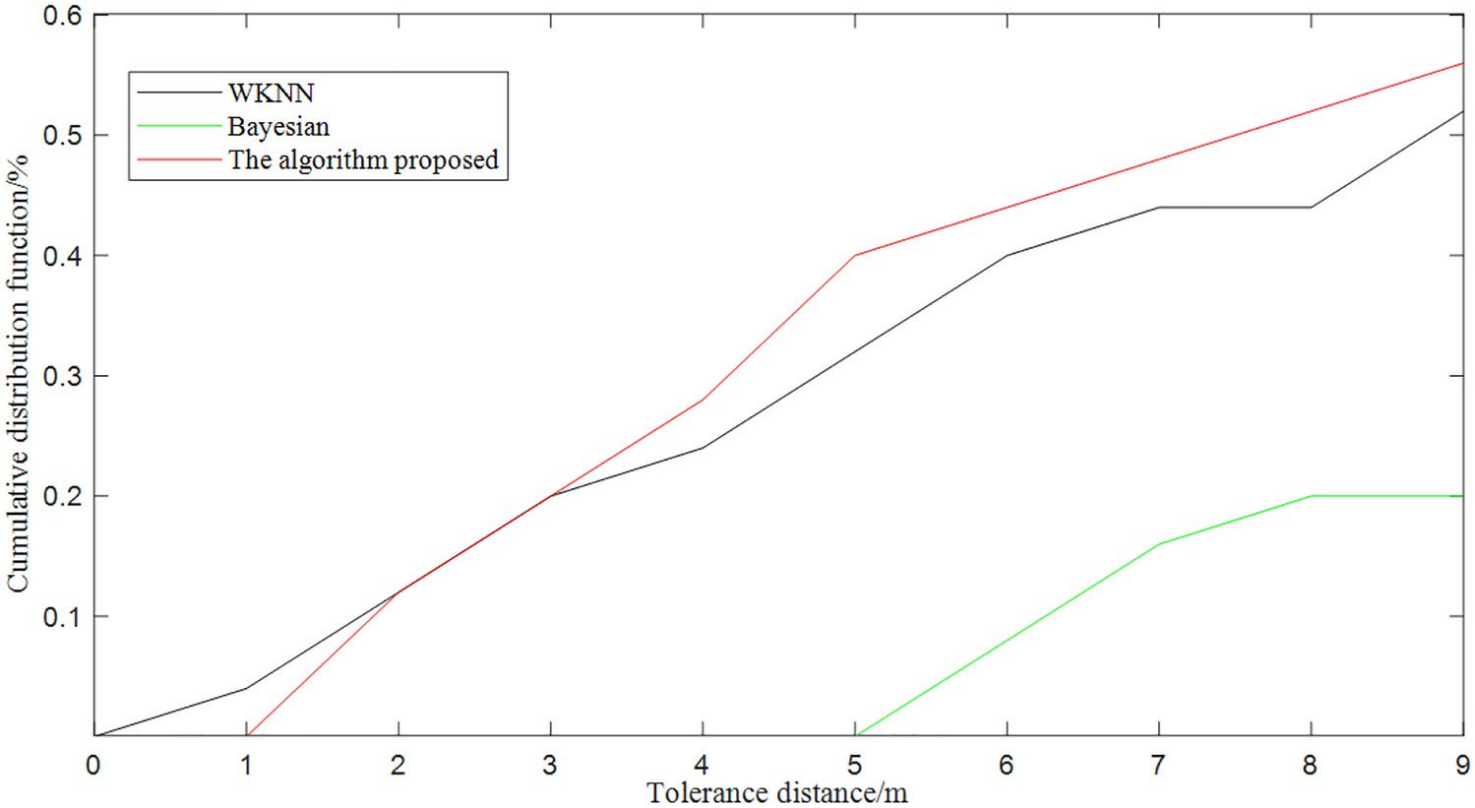
Error curves of localization results based on double clustering.

We evaluated it using the benchmark dataset (i.e. UJIndoorLoc dataset provided in^41^) and obtained the localization results in Fig. 15 and the cumulative distribution function in Fig. 16. The average positioning error can reach 7.0m at $50\%$, which is more effective compared to current clustering algorithms^42^.

## Conclusion

Future work can be carried out from the following aspects: (1) Real time collection of fingerprint point information during the subsequent update process of offline fingerprint database^43^ is a research focus. (2) Reduce the cost of offline fingerprint database collection.

## Supplementary Information

Supplementary Information.

Supplementary Information.

Supplementary Information.

Supplementary Information.

## Data Availability

Data is provided within the manuscript or supplementary information files

## References

[1] Shang, S. & Wang, L. Overview of wifi fingerprinting-based indoor positioning. *IET Commun.* **16**(7), 725–733 (2022).

[2] Qin, F., Zuo, T. & Wang, X. Ccpos: Wifi fingerprint indoor positioning system based on cdae-cnn. *Sensors* **21**(4), 1114 (2021). 33562754 10.3390/s21041114 PMC7915958

[3] Liu, F. et al. Survey on wifi-based indoor positioning techniques. *IET Commun.* **14**(9), 1372–1383 (2020).

[4] Oh, J. & Kim, J. Adaptive k-nearest neighbour algorithm for wifi fingerprint positioning, *ICT Express*, **4**(2), (2018).

[5] Singh, N., Choe, S. & Punmiya, R. Machine learning based indoor localization using wi-fi rssi fingerprints: An overview. *IEEE Access* (2021).

[6] Hoang, M. T., Yuen, B., Dong, X., Lu, T. & Reddy, K. Recurrent neural networks for accurate rssi indoor localization. *IEEE Internet Things J.* **PP**(99), 1–1 (2019).

[7] Yong, Y. F., Tan, C. K. & Tan, I. K. T. Smote for wi-fi fingerprint construction in indoor positioning systems. In *2021 IEEE International Performance, Computing, and Communications Conference (IPCCC)*, pp. 1–6, (2021).

[8] Du, X., Liao, X., Liu, M. & Gao, Z. Crcloc: A crowdsourcing-based radio map construction method for wifi fingerprinting localization. *IEEE Internet Things J.* **9**(14), 12 364-12 377 (2022).

[9] Ren, J., Wang, Y., Niu, C., Song, W. & Huang, S. A novel clustering algorithm for wi-fi indoor positioning. *IEEE Access* **PP**(99), 1–1 (2019).

[10] Lee, S. G. & Lee, C. “Developing an improved fingerprint positioning radio map using the k-means clustering algorithm,” In *2020 International Conference on Information Networking (ICOIN)*, (2020).

[11] D. B. N. A. B, J. H. A, V. T. T. A, & D. P. H. B, An effective random statistical method for indoor positioning system using wifi fingerprinting. *Future Generation Computer Systems* **109**, pp. 238–248, (2020).

[12] Liang, Y., Chen, S. & Dong, X. L. T. Fine-grained grid computing model for wi-fi indoor localization in complex environments. *J. Electron. Sci. Technol.* **22**(1), (2024).

[13] Ma, Z., Wu, B. & Poslad, S. A wifi rssi ranking fingerprint positioning system and its application to indoor activities of daily living recognition. *Int. J. Distrib. Sens. Netw.* **15**(4), 155014771983791 (2019).

[14] Cui, X., Wang, M., Li, J., Ji, M. & Chen, H. Indoor wi-fi positioning algorithm based on location fingerprint, *Mobile Netw. Appl.* **26**(4), (2021).

[15] Haider, A., Wei, Y., Liu, S. & Hwang, S. H. Pre- and post-processing algorithms with deep learning classifier for wi-fi fingerprint-based indoor positioning. *Electronics* **8**(2) (2019).

[16] Park, K. An efficient indoor positioning method based on wi-fi rss fingerprint and classification algorithm. *Sensors* **21**, (2021). 10.3390/s21103418 PMC8156841 34069023

[17] Jiusong, H., Dawei, L., Zhi, Y., & Hongli, L. Experimental analysis on weight -nearest neighbor indoor fingerprint positioning. *IEEE Internet of Things Journal*, (2018).

[18] Hosseini, K. S., Azaddel, M. H., Nourian, M. A. & Azirani, A. A. Improving multi-floor wifi-based indoor positioning systems by fingerprint grouping, In *International Conference on Internet of Things and Applications*, (2021).

[19] Shi, L. F. et al. A fusion algorithm of indoor positioning based on pdr and rss fingerprint. *IEEE Sens. J.* **PP**(23), 1–1 (2018).

[20] Zhang, H., Hu, B., Xu, S., Chen, B. & Jiang, B. Feature fusion using stacked denoising auto-encoder and gbdt for wi-fi fingerprint based indoor positioning. *IEEE Access* **PP**(99), 1–1 (2020).

[21] Ye, T. & Long, Z. A novel system for wifi radio map automatic adaptation and indoor positioning. *IEEE Trans. Vehic. Technol.* **PP**, 1–1, (2018).

[22] Yu, D. & Li, C. An accurate wifi indoor positioning algorithm for complex pedestrian environments, *IEEE Sens. J.* 21-21, (2021).

[23] Tao, Y. & Zhao, L. Aips: An accurate indoor positioning system with fingerprint map adaptation. *IEEE Internet Things J.* **PP**(99), 1–1 (2021).

[24] G. H. A. A, I. G. B. S. A, & J. V. a, Feature selection on database optimization for wi-fi fingerprint indoor positioning, *Proc. Comput. Sci.* **159**, 251–260, (2019).

[25] Maung, N. A. M., Lwi, B. Y. & Thida, S. An enhanced rss fingerprinting-based wireless indoor positioning using random forest classifier. In *2020 International Conference on Advanced Information Technologies (ICAIT)*, (2020).

[26] Zhu, Y., Luo, X., Guan, S. & Wang, Z. Indoor positioning method based on wifi/bluetooth and pdr fusion positioning. In *2021 13th International Conference on Advanced Computational Intelligence (ICACI)*. IEEE, pp. 233–238 (2021)

[27] Wang, X., Chen, Z., Zhang, S. & Zhu, J. Super-resolution based fingerprint augment for indoor wifi localization. In *GLOBECOM 2020 - 2020 IEEE Global Communications Conference*, (2020).

[28] Duan, Y., Lam, K. Y., Lee, V. C. S., Nie, W., Liu, K., Li, H. & Xue, C. J. Data rate fingerprinting: a wlan-based indoor positioning technique for passive localization. *IEEE Sens. J.* 1–1 (2019).

[29] Luo, M., Zheng, J., Sun, W. & Zhang, X. Wifi-based indoor localization using clustering and fusion fingerprint. In *2021 40th Chinese Control Conference (CCC)*, (2021).

[30] Jian, H. X. & Hao, W. Wifi indoor location optimization method based on position fingerprint algorithm, In *2017 International Conference on Smart Grid and Electrical Automation (ICSGEA)*, (2017).

[31] Torres-Sospedra, J. et al. Scalable and efficient clustering for fingerprint-based positioning. *IEEE Internet Things J.* **10**(4), 3484–3499 (2023).

[32] Sadhukhan, P., Dahal, K. & Das, P. K. A novel weighted fusion based efficient clustering for improved wi-fi fingerprint indoor positioning. *IEEE Trans. Wirel. Commun.* **22**, 4461–4474 (2023).

[33] Sadhukhan, P., Gain, S., Dahal, K., Chattopadhyay, S. & Wang, X. An efficient clustering with robust outlier mitigation for wi-fi fingerprint based indoor positioning. *Appl. Soft Comput.*, no. 3, p. 107549, (2021).

[34] Zhang, Y., Zhang, S., Li, R., Guo, D., Wei, Y. & Sun, Y. Wifi fingerprint positioning based on clustering in mobile crowdsourcing system. In *2017 12th International Conference on Computer Science and Education (ICCSE)*. IEEE, pp. 252–256. (2017).

[35] Pu, Q., Chen, Y., Zhou, M., Ng, J.K.-Y. & Cai, R. Bayesian meta-learning: Toward fast adaptation in neural network positioning techniques. *IEEE Internet Things J.* **11**(8), 14 924-14 937 (2024).

[36] Zhao, Z., Lou, Z., Wang, R., Li, Q. & Xu, X. I-wknn: Fast-speed and high-accuracy wifi positioning for intelligent sports stadiums. *Comput. Electr. Eng.* **98**, 107619 (2022).

[37] Wang, B., Liu, X., Yu, B., Jia, R. & Gan, X. An improved wifi positioning method based on fingerprint clustering and signal weighted euclidean distance. *Sensors* **19**(10), 2300 (2019). 31109054 10.3390/s19102300 PMC6567165

[38] Zhang, W. et al. A novel wifi indoor positioning strategy based on weighted squared euclidean distance and local principal gradient direction. *Sens. Rev.* **39**(1), 99–106 (2019).

[39] Singh, N., Choe, S. & Punmiya, R. Machine learning based indoor localization using wi-fi rssi fingerprints: An overview, *IEEE Access*, (2021).

[40] Hoang, M. T., Yuen, B., Dong, X., Lu, T. & Reddy, K. Recurrent neural networks for accurate rssi indoor localization. *IEEE Internet Things J.* **PP**(99), 1–1, (2019).

[41] Torres-Sospedra, J., Montoliu, R., Martinez-Uso, A., Avariento, J. P. & Huerta, J. Ujiindoorloc: A new multi-building and multi-floor database for wlan fingerprint-based indoor localization problems. *IEEE*, (2014).

[42] Sadhukhan, P., Dahal, K. & Das, P. K. A novel weighted fusion based efficient clustering for improved wi-fi fingerprint indoor positioning. *IEEE Trans. Wireless Commun.* **22**, 4461–4474 (2023).

[43] Serbouh, C., Njima, W. & Ahriz, I. Generative adversarial networks based data recovery for indoor localization. In *2024 IEEE Wireless Communications and Networking Conference (WCNC)*, pp. 1–6. (2024).

